# RNA-Seq Identifies Key Reproductive Gene Expression Alterations in Response to Cadmium Exposure

**DOI:** 10.1155/2014/529271

**Published:** 2014-05-27

**Authors:** Hanyang Hu, Xing Lu, Xiang Cen, Xiaohua Chen, Feng Li, Shan Zhong

**Affiliations:** Department of Medical Genetics, School of Basic Medical Science, Wuhan University, Wuhan 430071, China

## Abstract

Cadmium is a common toxicant that is detrimental to many tissues. Although a number of transcriptional signatures have been revealed in different tissues after cadmium treatment, the genes involved in the cadmium caused male reproductive toxicity, and the underlying molecular mechanism remains unclear. Here we observed that the mice treated with different amount of cadmium in their rodent chow for six months exhibited reduced serum testosterone. We then performed RNA-seq to comprehensively investigate the mice testicular transcriptome to further elucidate the mechanism. Our results showed that hundreds of genes expression altered significantly in response to cadmium treatment. In particular, we found several transcriptional signatures closely related to the biological processes of regulation of hormone, gamete generation, and sexual reproduction, respectively. The expression of several testosterone synthetic key enzyme genes, such as Star, Cyp11a1, and Cyp17a1, were inhibited by the cadmium exposure. For better understanding of the cadmium-mediated transcriptional regulatory mechanism of the genes, we computationally analyzed the transcription factors binding sites and the mircoRNAs targets of the differentially expressed genes. Our findings suggest that the reproductive toxicity by cadmium exposure is implicated in multiple layers of deregulation of several biological processes and transcriptional regulation in mice.

## 1. Introduction

Cadmium is an environmental and occupational toxic heavy metal that is widely used in industrial process and consumer products. The usual pattern of the nonoccupational cadmium intake is mainly from food, drinking water, and smoking [1], which caused several diseases by toxically targeting the lung, liver, kidney, bone, and the cardiovascular system as well as the immune and the reproductive system [2]. The multiple mechanisms involved in response to cadmium include modulating cell cycle, DNA repair process, DNA methylation status, altering gene expression, and several signaling pathways in carcinogenesis and other diseases [3–5].

It has been well documented that cadmium exposure leads to the impairment of male and female reproductive system both in human and animals. The high level cadmium in the serum and seminal fluid positively correlated to the azoospermia in the infertile Nigerian males [6]. In the female reproductive system, cadmium exposure leads to failure to ovulate, defective steroidogenesis, suppressed oocyte maturation, and failure of developmental progression in preimplantation embryo and implantation. Moreover, by using the established animal model, the cadmium exposure causing the reproductive system damage is associated with a series of abnormalities, including disruption of blood-testis barrier, testicular necrosis, disruption of blood-epididymis barrier, and abnormal sperm morphology [7]. In addition to reproductive system, recent study also indicated that prenatal cadmium exposure perturbs the vascular and immune system of the murine offspring [8, 9], implicating the role of cadmium exposure in offspring health. A number of mechanisms of reproductive toxicity of cadmium have been suggested, including ionic and molecular mimicry, interference with cell adhesion and signaling, oxidative stress, genotoxicity, and cell cycle disturbance [7]. Although the DNA microarray was employed to study the transcriptional gene alternation in cell lines and peripheral blood cells exposed to cadmium and identified several differentially expressed genes as well as signaling pathways [10, 11], the comprehensive understanding of the mechanisms that are responsible for the toxicity of chronic cadmium exposure in testis is still lacking.

In this study, we performed the RNA-seq to profile the alterations of gene expression in response to chronic cadmium exposure. By analyzing the transcriptome between the cadmium treated and untreated mice, we identified a number of transcriptional signatures, which provided mechanistic insight into the mechanism of how the male reproductive system is affected by chronic cadmium exposure.

## 2. Materials and Methods

### 2.1. Chemicals

Cadmium chloride (CdCl_2_) was purchased from Sigma Chemical Co. (St. Louis, MO).

### 2.2. Animals and Experimental Design

Thirty six-week-old male Chinese Kun Ming (KM) mouse weighing about 30–32 g were used in the experiment. The animals were obtained from Wuhan University Center for Animal Experiments/A3-Lab. All animals were housed in a laboratory-controlled environment (25°C, 50% humidity, and light : dark cycle 12 h : 12 h). The animals were permitted free access to food and drinking water* ad libitum*. The food for mice was purchased from Wuhan University Center for Animal Experiment. All animal experiments were carried out in strict accordance with the recommendations in the Regulations for the Administration of Affairs Concerning Experimental Animals of China. The protocol was approved by the Wuhan University Center for Animal Experiment (approved permit number: SCXK 2008-0004). All surgery was performed under sodium pentobarbital anesthesia, and all efforts were made to minimize suffering.

After acclimatization and one-week observation, we found the daily food consumption per mouse was about 6–8 g. Then all animals were randomly divided equally into three groups and every five mice were housed in a cage. To calculate the consumption of food containing cadmium, we make high-cadmium food as 0.3 mg CdCl_2_/g and low-cadmium food as 0.15 mg CdCl_2_/g. Then, each cage of high level cadmium-exposed group was supplied with 50 g high-cadmium food daily, and low level cadmium-exposed group was supplied with 50 g low-cadmium food as well. On the following day, we collected and removed the remaining food and residue and new 50 g food was given. By deducting the weights of remaining food and residue, we calculated that the food intake for cadmium treated mice was 6.5 ± 0.8 g. Therefore, the intake of cadmium in the high group was considered as 1.95 ± 0.24 g per mouse daily and in the low group was considered as 0.975 ± 0.12 g per mouse daily. Subsequently, all animals were treated with the according food for 6 months, in which the mice of control group were treated with the same amount of food without CdCl_2_. We chose these specific dose and period of cadmium exposure as our preliminary results showed that cadmium accumulation in serum and testis reached the highest value at six months, and the mice treated with such dose of cadmium did not show abnormalities or other health problems. Then the blood samples of 5 different mice in each group were randomly collected. Serum were separated by centrifugation from the blood samples above and stored at −80°C until assay for the cadmium concentration using the graphite furnace atomic absorption spectrometry (GFAAS) method. Testosterone in serum was measured using Testosterone Parameter Assay kit (R&D, USA).

### 2.3. RNA Isolation and Preparation for Next-Generation Sequencing

A total of 9 testis samples (three samples from each group) were selected for RNA isolation. Total RNA was isolated using Trizol Reagent (Invitrogen) according to the manufacturer's instructions. Then these RNA samples were sent to Analytical & Testing Center at Institute of Hydrobiology, Chinese Academy of Sciences (http://www.ihb.ac.cn/fxcszx/) for the verification of RNA integrity. Then one RNA sample from each group was collected for pair-ends transcriptome sequencing under the Illumina Genome Analyzer IIx platform. The sequencing data have been deposited in NCBI Sequence Read Archive (SRA, http://www.ncbi.nlm.nih.gov/Traces/sra/) with the accession number SRP032958.

### 2.4. Read Alignment with TopHat and RNASEQR

Raw data were mapped to the mouse reference genome (mm10 downloaded from UCSC) using TopHat (version 2.0.3) and RNASEQR (version 1.0.2) software, respectively [12, 13]. Prebuilt genomic indices were created by bowtie and provided to the alignment software for reads mapping. TopHat removes a few low-quality score reads then aligns the reads that are directly mapped to the reference genome. It then determines the possible location of gaps in the alignment based on splice junctions flanking the aligned reads and uses gapped alignments to align the reads that were not aligned by Bowtie in the first step. Compared with other alignment tools, RNASEQR takes advantages of annotated transcripts and genomic reference sequences to obtain high quality mapping result by the three sequential processing steps. It aligns the RNA-seq sequences to a transcriptomic reference firstly, then detects novel exons and identifies novel junctions using an anchor-and-align strategy finally. The output of Tophat can be the input of Cufflinks, while the output of RNASEQR can be converted as the input for Cufflinks using the convert_RNASEQRSAM_to_CufflinkSAM.py script.

### 2.5. Transcriptome Reconstruction

Aligned reads from TopHat and RNASEQR were assembled by Cufflinks (version 2.0.2), an* ab-initio *tancscriptome assembler that reconstructs the transcriptome based on RNA-seq reads aligned to the genome with a spliced read aligner. To obtain transcriptome assemblies from the aligned reads, we run Cufflinks with default parameters. After that, normalized expression levels were estimated and reported as FPKM (Fragments Per Kilobase of exon per Million fragments mapped). As several assembled transcripts were obtained from each sample, we used Cuffmerge to assemble them into a comprehensive set of transcripts for further downstream differential expression analysis.

### 2.6. Differential Expression Analysis

In order to determine the differentially expressed transcripts within the dataset, we used Cuffdiff, a separate program included in Cufflinks, to calculate expression in two or more samples and test the statistical significance of each observed change in expression between them. Cuffdiff reports numerous output files containing the results of the differential analysis of the samples, including genes and transcripts expression level changes, familiar statistics such as log_2_-fold change, *P* values (both raw and corrected for multiple testing), and gene-related attributes such as common name and genome location. The differentially expressed genes were clustered and visualized by TreeView [14]. Functional annotations of these genes were performed by the DAVID bioinformatics resources [15] and WEB-based GEne SeT AnaLysis Toolkit (WebGestalt) [16]. Downstream enrichment analyses such as TF binding sites in promoter regions and microRNA sites in 3′-UTRs were performed using Expander (version 6.0.5) [17], and the miRNA-target gene network was constructed by Cytoscape [18].

### 2.7. qPCR

qPCR analyses were performed to validate the results of RNA-seq. The reverse transcription is synthesized using RevertAidTM First Strand cDNA Synthesis Kit from Fermentas according to the manufacturer's instructions. The PCR primers were designed with Primer Premier 5.0 software and *β*-Actin was used as a reference gene. The primer sequences, melting temperatures, and product sizes are shown in Table 1. qPCR was performed on iQ5 Real Time PCR Detection System (Bio-Rad) (Bio-Rad, USA) using SYBR Green Realtime PCR Master Mix (TOYOBO CO., LTD, Japan) as the readout. Three independent biological replicates of the control and cadmium treated groups were included in the analysis and all reactions were carried out in triplicates. Data was analyzed by the 2-ΔΔCT method [19].

### 2.8. Statistical Analysis

Besides those statistical tools embedded in the bioinformatics software and resources, additional statistical analyses were performed using GraphPad Prism (Version 5.00). Cadmium treated groups were compared with the control groups by unpaired Student's* t*-test. *P* < 0.05 was considered statistically significant.

## 3. Results

### 3.1. Cadmium Accumulated in Mouse and Inhibited the Testosterone

**Figure 1:**
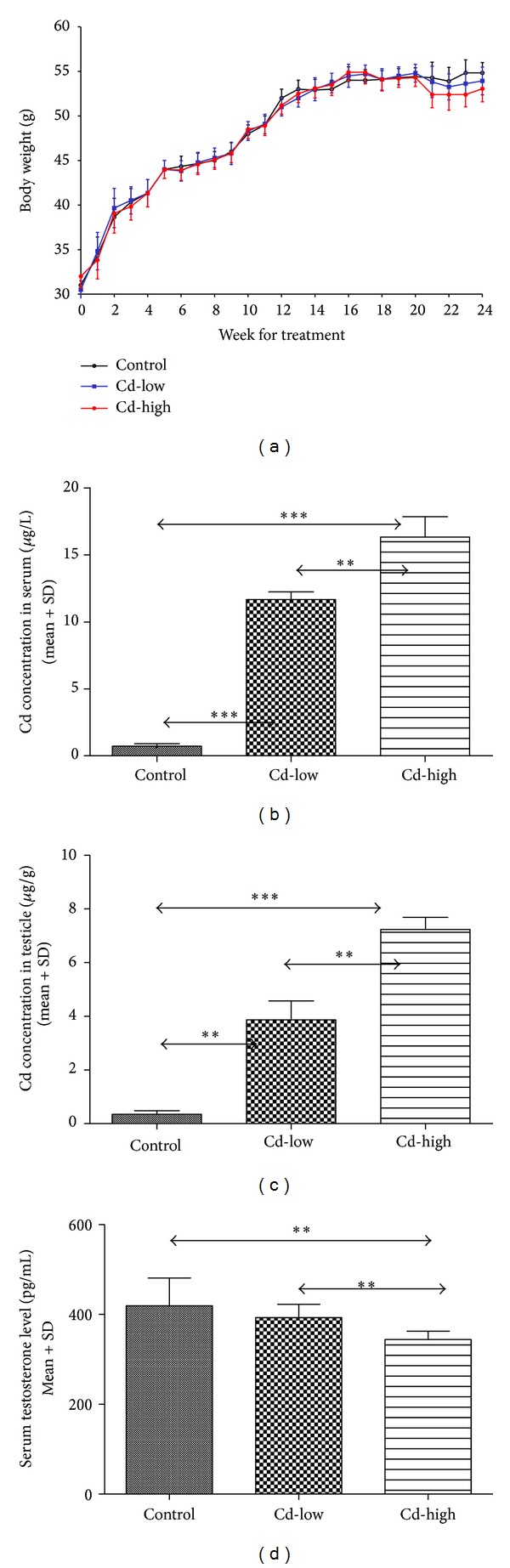
Body weights, level of cadmium in serum and testis, and serum testosterone. Mice were treated with different level of CdCl_2_ in their rodent chow for six months. (a) Body weights were measured per week during cadmium treatment. The level of accumulated cadmium in serum (b) and testis (c) were measured using the graphite furnace atomic absorption spectrometry (GFAAS) method. The levels of serum testosterone (d) were measured by ELISA. Data was shown as mean ± standard deviation (*n* = 5). (**P < 0.01, ***P < 0.001, Student's* t*-test).

After six-month cadmium-exposure treatment, all animals survived with the slight loss of body weight for the treated groups (Figure 1(a)) and did not show abnormalities or other health problems. We first tested the concentration of cadmium in serum and testis for each of the groups. As expected, cadmium concentration was significantly increased in proportion to the exposure level both in serum and testis (Figures 1(b)-1(c)). We examined the level of serum testosterone, which is essential for normal spermatogenesis and other reproductive processes. Results showed that cadmium exposure significantly reduced the level of serum testosterone in high dose cadmium treated mouse compared with the low and control mouse (Figure 1(d)).

### 3.2. The Next-Generation Transcriptome Sequencing

**Figure 2:**
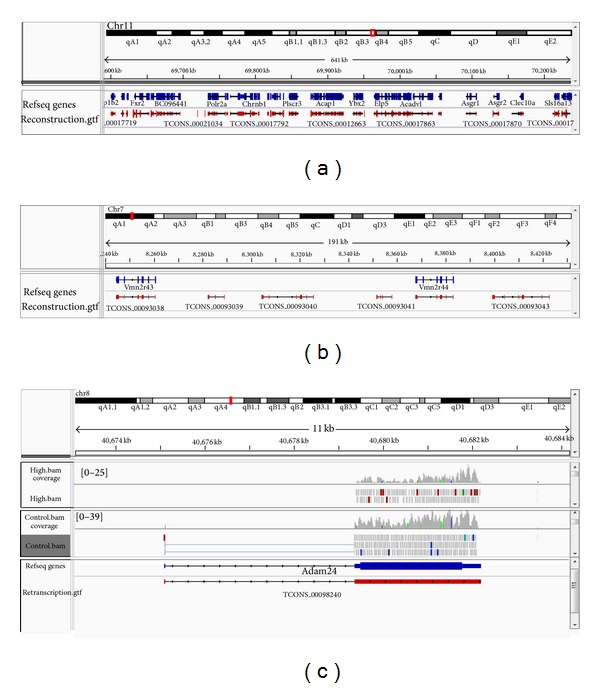
Testicular transcriptome reconstruction of RNA-seq under mouse reference annotation. (a) The genes on chr11:69,594,778–70,305,335 were reconstructed as examples. (b) The intergenic transcription was detected beyond the reference annotation. (c) Read coverage of Adam24 gene on chr8 was shown.

To determine the molecular events during cadmium exposure, we performed RNA-seq on testis samples of treated and untreated mice. Raw data of 80 million, 36-bp pair-ends reads were obtained and aligned to the mouse reference genome by TopHat and RNASEQR software [12, 13], resulting in, on average, 71% of the reads mapped to the reference genome with 57% unique mapped reads. Then, both the unique and multiple mapped reads were kept as Cufflinks can handle the multimapped reads by uniformly dividing each multimapped read to all of the positions it maps to and calculating initial abundance. Then the mapped reads were assembled for reconstructing transcriptome and estimating expression abundance by Cufflinks. The results of estimated normalized expression levels were reported as FPKM (Fragments Per Kilobase of exon per Million fragments mapped). Overall, a total of 27600 transcripts on average were successfully reconstructed from each group and mapped to the annotated genomic loci. For example, the genes on chr11:69,594,778–70,305,335 were reconstructed (Figure 2(a)) and the intergenic transcribed regions were pervasively detected compared with the mouse gene reference annotations (Figure 2(b)).  Figure 2(c) shows a representative example histogram of read coverage versus a genomic loci containing the adam24 gene.

### 3.3. Differentially Expressed Genes Analysis

**Figure 3:**
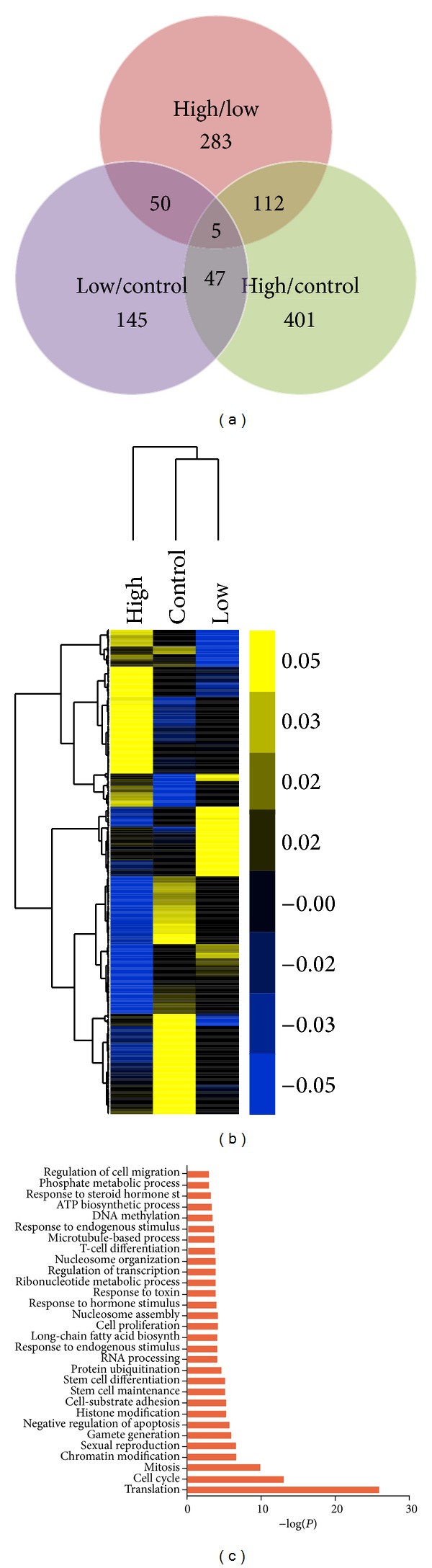
Cadmium modulated differentially expressed genes analysis. (a) The number of genes differentially expressed between the high level cadmium treated, low level cadmium treated, and control groups. (b) Hierarchical clustering analysis of gene expression profiles. Each column represents one mouse, and each horizontal line refers to a gene. Color legend is on the top-left of the figure. Yellow indicates genes with a greater expression relative to the geometrical means; blue indicates genes with a lower expression relative to the geometrical means. (c) Biological process Gene Ontology (GO) analysis of differentially expressed genes.

After reads mapping, transcripts assembling, and expression level calculating, we next sought to identify differentially expressed genes between samples with different treatment. By using Cuffdiff, two expression profiles were obtained from different mapping software. We then compared them and combined the overlapped differentially expressed genes. Genes with* q* value < 0.05 were considered to be differentially expressed. Finally, a total of 830 genes were identified as differentially expressed (Table S1 available online at http://dx.doi.org/10.1155/2014/529271). In detail, there were 283 differentially expressed genes between high and low groups (103 upregulated and 180 downregulated), 401 genes between high and control groups (137 upregulated and 264 downregulated), and 145 genes between low and control groups (61 upregulated and 84 downregulated), respectively (Figure 3(a)). All the differentially expressed transcripts were hierarchically clustered and the results showed that the distinct gene expression pattern was associated with cadmium exposure level, although the low and control groups exhibited a more similar expression pattern than the high group, probably because we used quite low dose of cadmium to treat the low group (Figure 3(b)).

In order to gain a comprehensive impact assessment of cadmium exposure on testicular gene expression, all biochemical pathways that altered in response to cadmium exposure were identified by comparing the ontology of all the genes differentially expressed between samples. Here, 373 differentially expressed genes annotated by UCSC and Ensembl of the 830 genes were performed with gene ontology enrichment analysis and functional classification. These genes were classified into several ontology categories according to their function in various biological processes (Figure 3(c)). Consistent with previous reports, some ontology categories that are implicated in cadmium toxicity to testis were confirmed in our study, such as genes involved in immunity, cell cycle, toxin, oxidation reduction, and metabolism [7].

Notably, the most enriched ontology category contains the genes associated with regulation of transcription. Genes that are involved in many classical signaling transduction pathways are modulated, such as Nfat5, E2f2, Fos, Junb, Notch1, and Stat4. In addition, we observed that abnormal epigenetic regulation occurred during cadmium exposure. Some of the differentially expressed genes involved in DNA methylation and histone modification are those with DNA methyltransferase, histone methyltransferase, acetyltransferase, or deacetylase activities, including Crebbp, Dmap1, Prdm9, Setd2, Prmt7, and Hdac2. Thus, both the transcriptional program and epigenetic patterns are supposed to be misregulated and implicated in cadmium caused reproductive toxicity.

Importantly, we also identified several novel and specific pathways modulated by cadmium exposure, including homeostasis of hormone (Table 2), gamete generation, and sexual reproduction (Table 3). Among these pathways, we noticed that similar functional categories shared the same differentially expressed candidate genes between them. The reproduction associated functional categories comprise 16 annotated genes, most of which were inhibited due to cadmium exposure. For example, Fndc3a, Dazl, Kitl, Tex15, and Zfx were downregulated with the fold changes ranging from −2.6 to −4.5. Since these genes are critical for spermatogenesis, germ cell development, or junctions between Sertoli cells [20–26], we speculated that cadmium induced testicular toxicity through targeting and downregulating these genes. The hormone related categories contained six genes, half of which were induced. In particular, the rest of three downregulated genes, named Star, Cyp11a1, and Cyp17a1, were specifically responsible for testosterone synthetic. The downregulation of these genes may contribute to the reduction of serum testosterone in response to cadmium exposure. Collectively, these findings suggested that cadmium impaired the reproductive process and spermatogenesisand also potentially modulated the normal hormone levels during the toxic response to cadmium in testis.

Besides, 9 KEGG signaling pathways were affected by cadmium exposure by mapping the differentially expressed genes to the KEGG database. Those modulated signaling pathways were comprised of ribosome, Alzheimer's disease, asthma, oxidative phosphorylation, focal adhesion, ECM-receptor interaction, C21-Steroid hormone metabolism, metabolic pathways, and prostate cancer (Table 4). Among these overrepresented pathways, according to functional hierarchies in KEGG, pathways of asthma are associated with the immune system. ECM-receptor interaction corresponds tosignaling molecules and interaction. Focal adhesion and ribosome are associated with cell communication and translation, respectively. Three modulated pathways, C21-Steroid hormone metabolism, oxidative phosphorylation, and metabolic pathways, are associated with metabolism, indicating the basal metabolism of cells in testis are affected. Altogether, our results reflected that multiple cellular and molecular mechanismsare modulated during cadmium exposure.

### 3.4. Validation of RNA-Seq Data by Quantitative Real Time PCR (qPCR)

**Figure 4:**
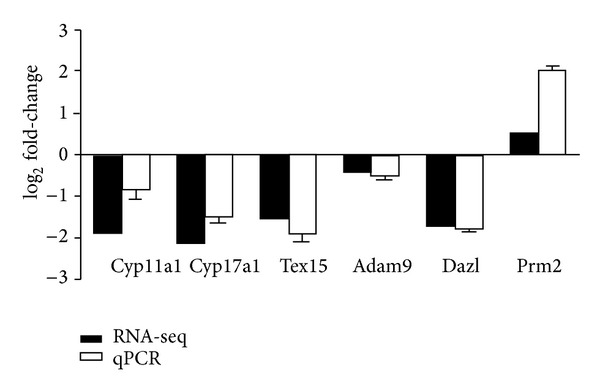
qPCR validation of the RNA-seq data. log_2_-fold change determined from the relative Ct values of six genes were compared to those detected by RNA-seq. Replicates (*n* = 3) of each sample were run and the Ct values averaged. All Ct values were normalized to beta-actin. *P* values of the Q-PCR data are 0.002 (Cyp11a1), 0.02 (Cyp17a1), 0.012 (Tex15), 0.014 (GLRX2), 0.008 (Adam9), 0.016 (Dazl), and 0.014 (Prm2), respectively.

To confirm the changes in gene expression observed by RNA-seq, we performed qPCR analysis on three reproductions (Prm2, Tex15, and Dazl), two hormones (Cyp11a1 and Cyp17a1) associated with functional categories genes, and a randomly selected gene named Adam9. qPCR results showed that these genes are significantly differentially expressed (*P* < 0.05) and exhibited the similar expression status compared to RNA-seq and conformed that Tex15, Dazl, Cyp11a1, and Cyp17a1 were inhibited by cadmium treatment (Figure 4).

### 3.5. Transcriptional and Posttranscriptional Control of Cadmium Modulated Genes

**Figure 5:**
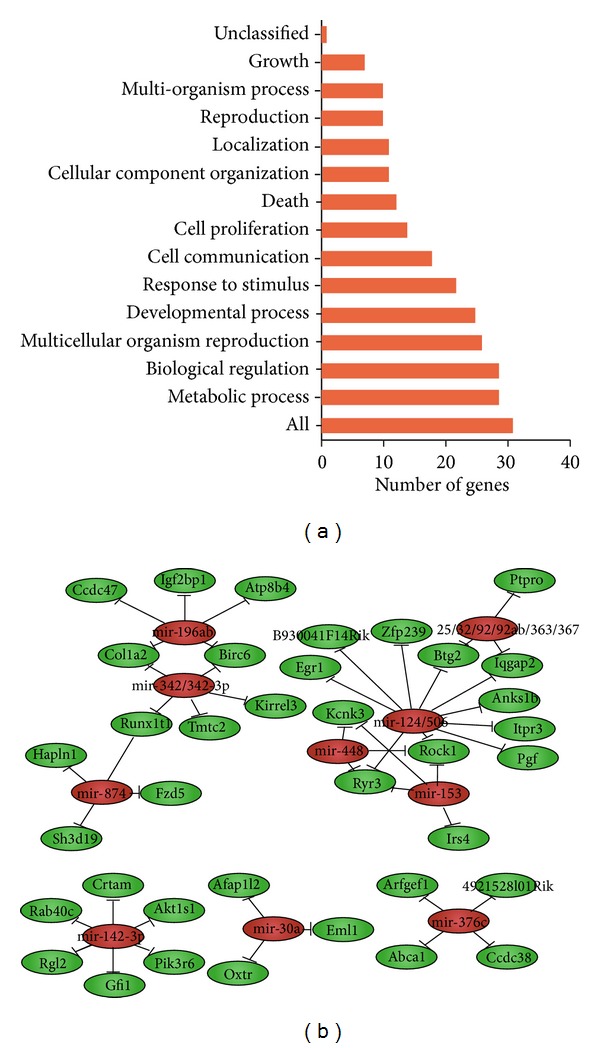
Transcriptional regulation analysis of the differentially expressed genes. (a) Biological process gene ontology analysis of the transcription factors that regulate the gene expression. (b) MicroRNA-target gene network. Red circles represent microRNAs; green circles represent the target genes.

In an effort to uncover the potential regulatory mechanism underlying the transcription of the cadmium modulated gene sets, we performed transcription factor binding sites analysis within the promoters and microRNA targets analysis of the cadmium modulated genes. Promoter regions for positions of −1000–+200 of the TSS across the cadmium modulated genes were predicted for the binding sites enrichment of several transcription factors (*P* < 0.05, Bonferroni test) (Table 5). Gene ontology analysis of these transcription factors revealed that they were involved in multiple biological processes containing regulation of cell cycle, hormone secretion, organ morphogenesis, reproductive process, neurogenesis, and response to stimulus, which were in accordance with the biological processes associated with the differentially expressed genes regulated by these transcription factors (Figure 5(a)).

We next performed microRNA targets analysis of the differentially expressed genes for further investigating the posttranscriptional control of them. A total of 10 microRNAs were identified as significantly enriched at 3′-UTR region of the differentially expressed genes (FDR < 0.05) (Table 6). By evaluating the microRNAs-Target-Network generated from the above information, it is implicated that a number of altered genes expression in this study may be regulated by the common microRNAs (Figure 5(b)), indicating their potential roles in regulating the reproductive process in response to cadmium exposure.

## 4. Discussion

Cadmium has been suggested to be anenvironmental and occupational toxic heavy metal that causes several diseases and toxically targets the lung, the liver, the kidney, the bone, the cardiovascular system, the immune system, and the reproductive system [2]. Here, in order to uncover the exclusive molecular mechanism of the mouse reproductive toxicity caused by chronic cadmium exposure, we simulated the conditions of cadmium pollution in human by treating the mouse with different doses of cadmium for 6 months. We observed cadmium exposure significantly reduced the level of serum testosterone. As a member of androgens, testosterone brings about its biological functions through associations with androgen receptor (AR), leading to AR transactivation which then results in the modulation of AR downstream gene expressions [27]. While the difference of testosterone seems to be small, such difference may lead to significantly downstream effects through the cascade signal transduction and transcriptional regulation of many genes by androgen receptor.

We next used RNA-seq to analyze the transcriptome of mouse testis affected by cadmium. We found a total of 830 genes and transcripts that were differentially expressed. Gene Ontology analysis revealed that these genes were enriched in several biological processes, in which the genes related to the reproductive process were paid more attention. For example, Fndc3a was reported to be required for adhesion between spermatids and Sertoli cells during mouse spermatogenesis [20]. Loss of Tex15 function causes early meiotic arrest in male mice, and Tex15-deficient spermatocytes exhibit a failure in chromosomal synapsis and DNA double-strand breaks are impaired [21]. In human, the deletion of DAZ cluster is associated with azoospermia and oligospermia in 5–10% of infertile men [22], and disruption of the Dazla gene leads to loss of germ cells and complete absence of gamete production [23]. In mouse, Dazl is required for embryonic development and survival of XY germ cells [24]. Kitl mutant mice exhibited reduced testis size due to aberrant spermatogonial proliferation, affecting the formation of tight junctions between Sertoli cells during postnatal development [25]. The zinc-finger proteins ZFX mutant mice were smaller and less viable and had fewer germ cells than wild-type mice [26]. Altogether, we supposed that the cadmium could cause the impairment of spermatogenesis and testicular toxicity through the repression of these genes. In addition, we identified six hormone related genes that were modulated by cadmium. We observed the decrease of expression for Star, Cyp11a1, and Cyp17a1, which were in accordance with a previous study [28]. As these genes encode the primarily testosterone synthetic enzymes, it is likely that the cadmium perturbed the spermatogenesis through repressing the synthesis of testicular testosterone as well. Combined with other modulated functional categories such as immunity, cell cycle, transcription, epigenetic regulation, and metabolism, the molecular mechanisms of cadmium caused male reproductive toxicity are implicated in multiple layers of deregulation of several biological processes.

Further, we computationally analyzed the transcriptional and posttranscriptional control of the differentially expressed genes. We found several transcriptional factors were enriched with the binding sites at the promoter regions of some gene sets. These binding events should be verified by further ChIP experiments. While these transcriptional factors were unable to be detected as statistically significantly differentially expressed between the samples, it is likely that the slight change of expression ultimately led to the significant expression change of their targets. We also predicted the microRNAs with the binding possibility of some sets of cadmium modulated genes. We identified 10 microRNAs targeted to the differentially expressed genes, the regulatory roles of which in testis response to cadmium could be explored by their expression patterns and the gain- or loss-of-function studies in the future.

In summary, our study demonstrated that many genes in testis were modulated due to chronic cadmium exposure. In particular, aside from the genes related to the functional categories previously reported, we identified novel pathways and the potential transcriptional regulatory mechanism on the cadmium modulated genes. These findings provide evidence for the elucidation of the molecular mechanism linking the chronic cadmium exposure to the impairment of male reproductive system and the clues for future studies of potential biomarkers and therapeutic targets for cadmium exposure.

## Supplementary Material

Table S1. Differentially expressed genes modulated by cadmium.

## Figures and Tables

**Table 1:** Primers used for qPCR validation.

Gene	Primer sequence (5′-3′)	Target size (bp)	Tm (°C)
Actin, beta	Forward: CTGTCGAGTCGCGTCCACCC Reverse: ACATGCCGGAGCCGTTGTCG	128	59

Cyp11a1	Forward: AGATCCCTTCCCCTGGTGACAATG Reverse: CGCATGAGAAGAGTATCGACGCATC	192	60

Cyp17a1	Forward: CCAGGACCCAAGTGTGTTCT Reverse: CCTGATACGAAGCACTTCTCG	250	59

Prm2	Forward: CAAGAGGCGTCGGTCA Reverse: TGGCTCCAGGCAGAATG	167	59

Tex15	Forward: ATTTGAGTGGCACAGAC Reverse: AGTATTGGGATTTGGAG	194	59

Adam9	Forward: CGCTTAGCAAACTACCTG Reverse: TTCCCGCCACTGAACAA	147	59

Dazl	Forward: GGAGGCCAGCACTCAGTCTTC Reverse: AGCCCTTCGACACACCAGTTC	184	60

**Table 2:** Regulation of hormone level related genes.

Gene symbol	Description	Fold change	*Q* value
Adh1	Alcohol dehydrogenase 1 (class I)	3.806345	0.005
Cyp11a1	Cytochrome P450, family 11, subfamily a, polypeptide 1	−7.197441	5.57*E* − 08
Cyp17a1	Cytochrome P450, family 17, subfamily a, polypeptide 1	−4.438219	0.0001
Ren1, Ren2	Renin 1 structural; similar to renin 2 tandem duplication of Ren1; renin 2 tandem duplication of Ren1	7.678866	0.016
Retsat	Retinol saturase (all trans retinol 13, 14 reductase)	4.603697	0.025
Star	Steroidogenic acute regulatory protein	−5.377734	5.57*E* − 05

**Table 3:** Regulation of reproductive process related genes.

Gene symbol	Description	Fold change	*Q* value
Adam24	A disintegrin and metallopeptidase domain 24 (testase 1)	−2.872645	0.038
Adam25	A disintegrin and metallopeptidase domain 25 (testase 2)	−2.736529	0.048
Adam26a	A disintegrin and metallopeptidase domain 26A (testase 3)	−3.429332	0.023
Dazl	Deleted in azoospermia-like	−3.38705	0.01
Fndc3a	Fibronectin type III domain containing 3A	−2.636543	0.044
Kitl	Kit ligand	−4.289398	0.044
Prm2	Protamine 2	1.169034	0
Qk	Similar to Quaking protein; quaking	−3.974115	0.006
Sycp2	Synaptonemal complex protein 2	−2.977031	0.031
Tex15	Testis expressed gene 15	−2.926283	0.0298951
Txndc3	Thioredoxin domain containing 3 (spermatozoa)	−2.973916	0.017
Zbtb16	Zinc finger and BTB domain containing 16	−6.408031	0.011
Zfp37	Zinc finger protein 37	−3.430625	0.004
Zfx	Zinc finger protein X-linked; similar to zinc finger protein	−4.58343	0.027347
Zan	Zonadhesin	−3.605458	0.00578172

**Table 4:** Modulated KEGG pathways.

Pathway name	Number of genes	Corrected *P* value
Ribosome	17	5.54*E* − 12
Alzheimer's disease	11	0.0246
Asthma	2	0.0486
Oxidative phosphorylation	9	0.0419
Focal adhesion	10	1.73*E* − 4
ECM-receptor interaction	7	1.15*E* − 4
C21-steroid hormone metabolism	2	0.0106
Prostate cancer	6	0.00868
Metabolic pathways	37	0.0059

**Table 5:** Enrichment of transcription factors across the promoter regions of differentially expressed genes.

Transcriptional factors	Number of genes	Corrected *P* value
ETF	193	4.37*E* − 16
NKX3A	178	0.007
Nrf-1	170	6.53*E* − 4
HMGIY	140	0.009
SRY	328	3.49*E* − 4
ZF5	199	2.05*E* − 7
FOXJ2	153	7.62*E* − 5
OCT-1	229	1.12*E* − 4
E2F-1	226	2.24*E* − 7
LUN-1	35	0.043
FOXP1	319	7.31*E* − 7
AP2	169	0.014

**Table 6:** MicroRNAs enriched at 3′-UTR region of the differentially expressed genes.

MicroRNA	Number of targeted genes	FDR
Mir-142-3p	6	0.001
Mir-342/342-3p	5	0.0015
Mir-196ab	5	0.0025
Mir-874	4	0.003
Mir-124/506	5	0.0135
Mir-30a	3	0.0075
Mir-124/506	11	0.0205
Mir-153	4	0.0265
Mir-25/32/92/92ab/363/367	3	0.0195
Mir-448	4	0.006
